# Solid-State [2+2] Photodimerization and Photopolymerization of α,ω-Diarylpolyene Monomers: Effective Utilization of Noncovalent Intermolecular Interactions in Crystals

**DOI:** 10.3390/molecules16010119

**Published:** 2010-12-28

**Authors:** Yoriko Sonoda

**Affiliations:** Photonics Research Institute, National Institute of Advanced Industrial Science and Technology (AIST), Higashi 1-1-1, Tsukuba, Ibaraki 305-8565, Japan; E-Mail: y.sonoda@aist.go.jp; Tel.: +81-29-861-6390; Fax: +81-29-861-4673.

**Keywords:** ring-substituted diphenylhexatriene, cyclobutane, [2+2] photocycloaddition, hydrogen bond, stacking arrangement

## Abstract

[2+2] Photocycloaddition of olefins is a very useful reaction in synthetic organic chemistry to obtain cyclobutane-containing molecules, which are almost inaccessible by other methods. The reaction, when performed in the crystalline state, occurs more efficiently and selectively than in homogeneous solution due to tight and regular molecular arrangement in the crystal state. Despite numerous examples for the solid-state [2+2] photodimerization of monoenes, however, it is still a challenge to prepare not only dimers but also higher oligomers and polymers from conjugated polyenes, which have multiple reactive double bonds in a molecule. In our recent studies of the solid-state photoreactions of α,ω-diarylpolyenes, noncovalent intermolecular interactions in crystals were effectively utilized to prealign molecules in stacking arrangements, suitable for the [2+2] reaction. With appropriate ring-substituents, [2+2] photodimerization and photopolymerization of the polyenes took place, although the degree of polymerization was relatively low. This review will describe the details of these reactions.

## 1. Introduction

[2+2] Photocycloaddition is one of the best known reactions of olefins in solid-state organic photochemistry [1,2,3,4,5,6,7,8,9,10,11,12,13,14,15,16]. By using the reaction, cyclobutane molecules, almost inaccessible by other synthetic methods, are easily obtained in high or at least reasonably good yields. The reaction, when performed in the crystalline state, occurs more efficiently and selectively than in homogeneous solution due to the tight and regular molecular arrangement found in crystals. It is often observed that the photoproducts in the solid state are entirely different from those in solution. Further, it has recently attracted increasing attention as one of the best suited reactions for 'green chemistry', because the reaction is 'solvent-less' in nature [14,15,16,17]. Even for two-component reactions, the mixing and grinding the reactant solids induce 'mechanochemical self-assembly' to lead high reaction efficiency without using any solvents [18,19,20,21,22]. In addition, only light irradiation is needed for the reaction to take place. No other energy supply such as heating equipment is required, as the photoreaction usually proceeds at or below room temperature. 

During 1960-1970s, Schmidt and co-workers thoroughly and systematically studied the solid-state [2+2] photocycloaddition of cinnamic acids (CAs) [1,2,23,24,25]. From crystallographic investigations 'topochemical rules', which connect the configuration of the product and crystal structure of the reactant, were revealed [6,9,23]. It is well-known that for topochemical [2+2] photocycloaddition, the distances between the potentially reactive double bonds should be less than *ca.* 4.2 Å ('Schmidt's rule') [1,2,25]. Since the pioneering work of Schmidt *et al.*, numerous examples of the solid-state [2+2] photo-dimerization of olefins, mostly monoenes, have been reported. Up to now, a number of strategies have been employed to prealign molecules in stacking arrangements favorable for the [2+2] reaction. These include intramolecular substitution to control intermolecular interactions between reactant olefins in crystals [11,26,27,28,29,30,31,32], inclusion within host structures [11,14,15,16], and cocrystallization with hydrogen-bond templates [33,34,35]. Coordination to a metal is also used to preorganize molecules for thereaction [36,37]. The use of tetranuclear rectangle macrocycles is an interesting new approach [38,39,40].

The [2+2] cycloaddition of molecules having two or more potentially reactive double bonds, such as diolefins and conjugated polyenes, can be a polymerization reaction. As for the reactions of aromatic diolefins, the [2+2] photopolymerization of distyrylpyrazines and phenylene diacrylates took place topochemically to afford highly crystalline polymers [41,42]. The reaction is known as 'four-center type photopolymerization'. For conjugated polyenes, however, it is still a challenge to prepare not only dimers but also higher oligomers and polymers. This is probably due to highly flexible polyenic chains even in the solid state. Although the hydrogen-bond templates have been successfully used to prepare ladder-shaped [2+2] dimers from conjugated dienes and trienes (in 100% yield), the reactions did not afford higher molecular weight (MW) products [33,34,35].

**Figure 1:**
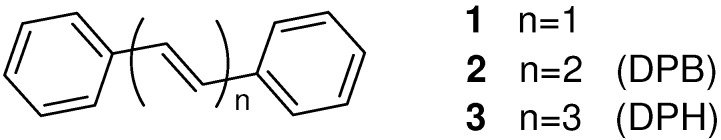
Chemical structures of α,ω-diphenylpolyenes **1**-**3**.

In our recent studies on the solid-state photoreactions of ring-substituted α,ω-diphenylpolyenes (Figure 1), noncovalent intermolecular interactions in crystals were effectively utilized to prealign molecules in stacking arrangements suitable for the [2+2] reaction. Unsubstituted parents of this class of molecules were all photochemically inert due to unfavorable crystal packing for the reaction. With appropriate substituents, however, [2+2] photodimerization and photopolymerization of the polyenes took place in the crystalline state. This review will mainly describe the photoreactions of ring-substituted (*E*,*E*,*E*)-1,6-diphenyl-1,3,5-hexatrienes (**3**; DPHs), and compare them with those of ring-substituted (*E*)-CAs and (*E*,*E*)-1,4-diphenyl-1,3-butadienes (**2**; DPBs).

## 2. [2+2] Photodimerization and Photopolymerization of α,ω-Diarylpolyenes

Unsubstituted α,ω-diphenylpolyenes, (*E*)-stilbene **1**, (*E*,*E*)-DPB, and (*E*,*E*,*E*)-DPH, were all photochemically stable in the solid state [43,44,45]. The molecules in the crystal structure of(*E*,*E*)-DPB [46,47] were arranged in a 'herringbone' pattern [48,49]. The nonparallel arrangement of double bonds of adjacent molecules was obviously unsuitable for the [2+2] cycloaddition. Also in (*E*,*E*,*E*)-DPH crystal, the molecular arrangement was unfavorable for the reaction, and the double bond distance for the nearest stacking molecules was 7.730 Å (= *a*) [50]. However, introduction of appropriate ring-substituents into the benzene rings of DPB and DPH led to π-stacked arrangements in crystals favorable for the [2+2] reaction.

### 2.1. Cyano Substitution

**Scheme 1:**
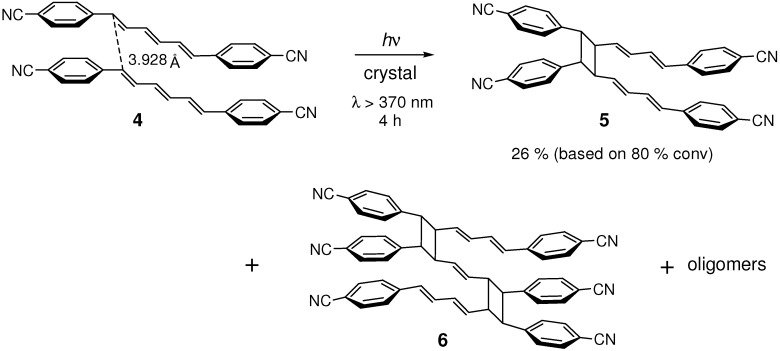
[2+2] Photodimerization and photopolymerization of **4**.

4,4'-Dicyano-substituted (*E*,*E*,*E*)-DPH **4** underwent crystalline-state [2+2] photocycloaddition at the terminal double bonds of the triene to give mirror-symmetric dimer **5**, trimers (**6**; the most plausible structure of the main trimer) and oligomers (Scheme 1) [45]. In the solid state, no *Z*-*E* geometrical photoisomerization was observed, in contrast to highly efficient and selective *EEE*→*ZEE* isomerization in diluted solution [51,52].

**Figure 2:**
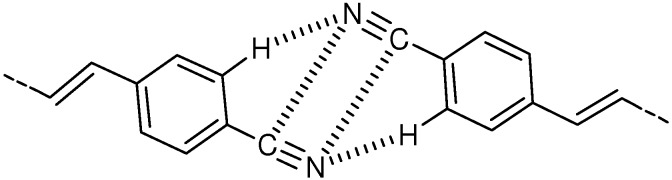
CH···N hydrogen bonding and CN···CN dipole-dipole interactions incrystal **4**.

In the crystal structure [53], molecules were linked by CH···N hydrogen bonding [54,55,56,57,58] and CN···CN dipole-dipole interactions [58] to make a tape (Figure 2). The tapes were further linked by CH···N hydrogen bonds to make a sheet. The molecules were π-stacked with the distance between the reactive double bonds of 3.928 Å, reasonably close each other. Thus the double bond distance of 7.73 Å in unsubstituted DPH [50] largely decreased to 3.93 Å in **4**, indicating the remarkable effect of cyano substitution. Considerably strong face-to-face interactions between π-orbitals of the molecules were evidenced by the observation of excimer fluorescence from the crystal [45,59]. The [2+2] photoreaction of **4** is considered to proceed *via* the excimer(s).

Interestingly, the very similar structural motif shown in Figure 2 has been observed in the crystal structure of 4-cyano-substituted (*E*)-CA [58]. The centro-symmetric carboxylic dimers of 4-cyano CA were organized into molecular sheets utilizing CN···CN dipole interactions and weaker forces such as CH···N and CH···O hydrogen bonds. The molecules were stacked with the double bond center-to-center distance of *ca.* 3.7-3.8 Å. Despite a large offset for the double bonds of stacking two molecules, the crystal was [2+2] photoreactive to give a mirror-symmetric dimer in 94% yield.

### 2.2. Formyl Substitution

**Scheme 2:**
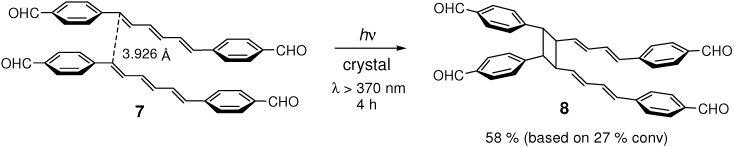
[2+2] Photodimerization of **7** in the crystalline state.

**Scheme 3:**
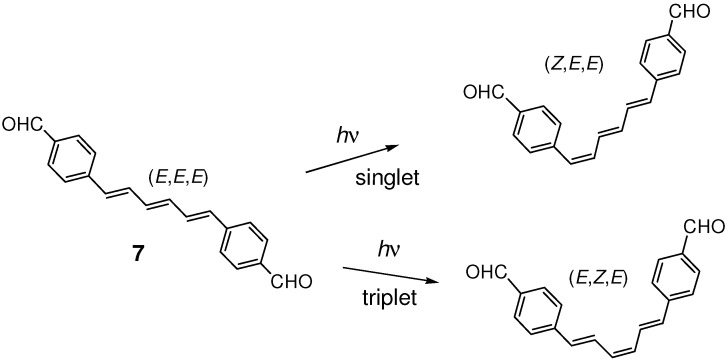
Regioselective *Z*-*E* photoisomerization of **7** in solution.

4-Formyl substitution on the benzene rings of (*E*,*E*,*E*)-DPH also induced the [2+2] photocycloaddition of hexatriene double bonds. Irradiation of **7** gave mirror-symmetric dimer **8** (Scheme 2) [45]. Crystal **7** was less reactive than **4** and the yield of higher oligomers than dimer was low. In diluted solution, **7** underwent efficient *Z*-*E* photoisomerization, whose regioselectivity (*EEE*→*ZEE* *vs*. *EEE*→*EZE*) depended on the spin multiplicity of the excited states involved in the reaction [60,61,62] (Scheme 3). While in the solid state, no *Z*-*E* isomerization was observed.

CH···O-type hydrogen bonds [57,63,64,65,66,67,68] were observed between the formyl groups of the neighboring molecules in crystal [53], in agreement with the results of IR and ^13^C CP/MAS NMR spectral measurements [45]. Molecules were π-stacked with the distance between the two reactive double bonds of 3.926 Å. As in **4**, the solid-state excimer fluorescence was observed in **7**, indicative of the presence of intermolecular π-orbital interactions of considerable strength [45,59].

4-Formyl-substituted (*E*)-CA also underwent [2+2] cycloaddition in the solid state to give a mirror-symmetric photodimer in a quantitative yield [69,70,71]. The molecules formed centro-symmetric carboxylic dimers, which piled up to form a plane-to-plane parallel stack. Although the shortest intermolecular distance between the reactive double bonds (4.825 Å) was significantly longer than the Schmidt's criterion of 4.2 Å, the plane-to-plane perpendicular distance between reactive molecules (3.388 Å) was fairly short, thus making the molecule [2+2] photoreactive [72]. 

As seen, the cyano and formyl substitution was both effective to prealign molecules for the [2+2] cycloaddition of (*E*,*E*,*E*)-DPH. It should be noted that molecules in the photoreactive crystals **4** and **7** are joined through relatively weak intermolecular interactions such as CH···N and CH···O hydrogen bonds [45].

### 2.3. Nitro Substitution

**Scheme 4:**
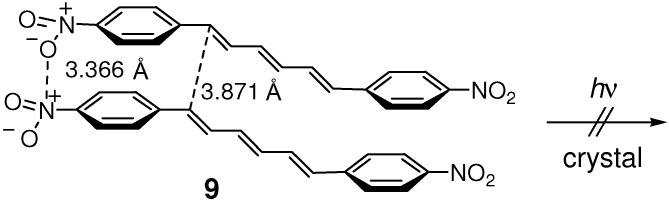
Stacking two molecules in photostable crystal **9**.

As in **7**, weak CH···O-type hydrogen bonds [68,73,74] were observed in the crystal structure of **9** (Scheme 4) [75]. The molecular sheets formed by the CH···O hydrogen bonds were linked by intermolecular N···O dipole interactions and aromatic π-π stacking interactions into a three-dimensional framework. The double bond distance for the stacking molecules was 3.871 Å, similar to or even slightly shorter than those for the [2+2] photoreactive crystals of **4** and **7**. Contrary to expectation, however, **9** was photochemically very stable in the crystalline state [45,76] as in solution [52].

Consistently, unlike **4** and **7**, the solid-state fluorescence of **9** originated not from excimeric but from monomeric species [76]. This indicates the absence of strong π-orbital-π-orbital interactions between the stacking molecules in the excited state, although they are in close proximity in the ground state. 

### 2.4. Alkoxy-Nitro (Donor-Acceptor) Substitution

**Scheme 5:**
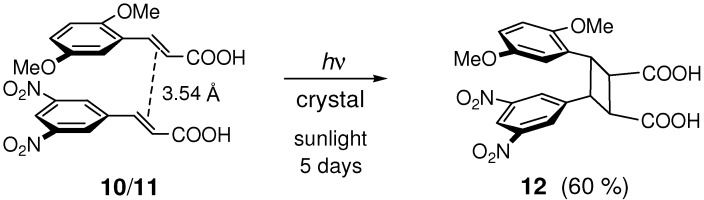
[2+2] Photocycloaddition of **10** and **11** in 1:1 cocrystal **10**/**11**.

Cocrystallization of 2,5-dimethoxy- and 3,5-dinitro-substituted (*E*)-CAs, **10** and **11** respectively, led to π-stacked molecular arrangements as a result of OH···O and CH···O hydrogen bonding, and donor-acceptor charge transfer (CT)-type stacking interactions [77,78]. The resulting 1:1 cocrystal **10**/**11** photoreacted to give **12** (Scheme 5).

Unfortunately, however, an attempt to cocrystallize 4,4'-dinitro DPH **9** and 4,4'-dimethoxy-substituted (*E*,*E*,*E*)-DPH was unsuccessful; from the 1:1 mixture of the two derivatives in acetonitrile, only single crystals of **9** were grown out from the solution.

**Scheme 6:**
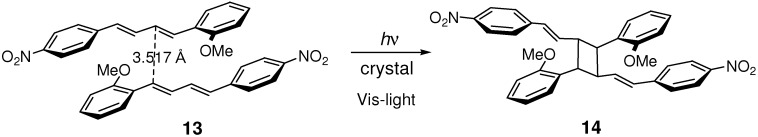
[2+2] Photodimerization of **13**.

**Scheme 7:**
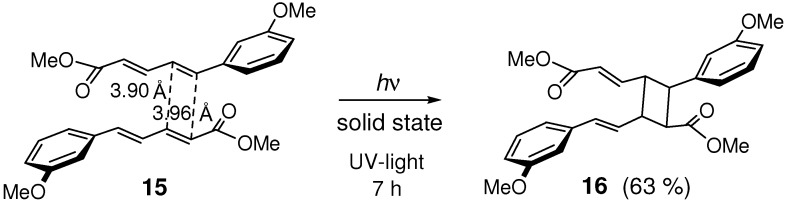
[2+2] Photodimerization of **15**.

The crystal of (*E*,*E*)-1-(2-methoxyphenyl)-4-(4-nitrophenyl)-1,3-butadiene **13** was irradiated to give head-to-tail dimer **14** (Scheme 6) [79]. Similarly, irradiation of donor-acceptor-substituted (*E*,*E*)-diene ester **15** gave dimer **16** (Scheme 7) [80]. In the crystal structure of **15**, the double bonds that reacted to form **16** were close to one another with the distances of 3.9-4.0 Å, and were arranged in a head-to-tail manner with only a slight offset.

**Figure 3:**
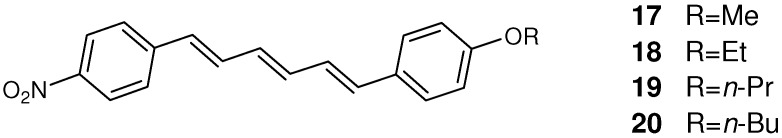
Chemical structures of alkoxy-nitro-substituted (*E*,*E*,*E*)-DPHs **17**-**20**.

For a series of (*E*,*E*,*E*)-1-(4-alkoxyphenyl)-6-(4-nitrophenyl)-1,3,5-hexatrienes **17**-**20** (Figure 3), the relationship between crystal structure and photophysical/photochemical properties was systematically investigated [81]. 

**Figure 4:**
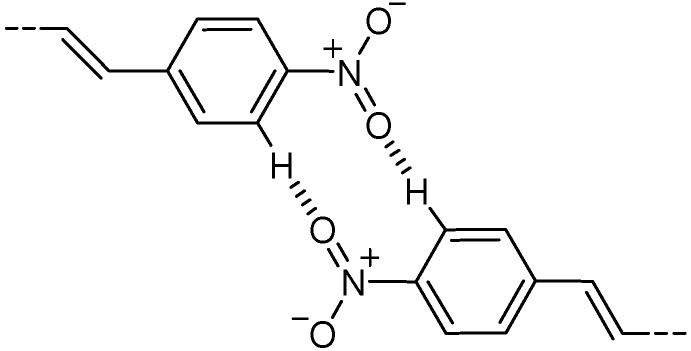
A hexagonal pattern formed by CH···O hydrogen bonds in crystal **20**.

**Scheme 8:**
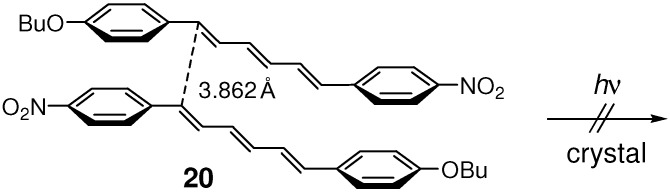
Stacking two molecules in photostable crystal **20**.

In the crystal structure of **20**, the O atom of the nitro group was in close contact with a neighboring H atom of the nitrophenyl ring at the *ortho* position relative to the nitro group to form a weak CH···O hydrogen bond [68,73,74]. A pair of this type of hydrogen bonds formed a hexagonal pattern between adjacent two molecules to make a tape (Figure 4) [55,57,73]. The molecular tapes were π-stacked with the double bond distance of 3.862 Å (Scheme 8). Also, molecules in crystal **18** were shown by powder X-ray diffraction (XRD) analysis to be arranged in a π-stacked fashion. While in crystals **17** and **19**, molecules were arranged in a herringbone pattern. Therefore at least **18** and **20** were expected to be [2+2] photoreactive. Actually, however, **17**-**20** were all photochemically stable in the solid state. The results were consistent with the fact that all four molecules exhibited solid-state fluorescence of monomeric origin but no excimer fluorescence. They underwent *Z*-*E* photoisomerization in low polar solvents [82], but did not isomerize in the crystalline state.

Thus, as a consequence of attractive CT interactions, the donor-acceptor (*E*,*E*)-DPB **13** and related molecule **15** were organized into π-stacked structures. The resulting crystals were [2+2] photoreactive, as expected. For donor-acceptor (*E*,*E*,*E*)-DPHs **17**-**20**, on the other hand, the ring-substitution was not effective in all cases to steer stacking molecular arrangements. 

The packing patterns of molecules (*i.e.*, herringbone and π-stacked) depended clearly on a slight difference in molecular structure such as the alkoxy chain length. This suggests that the two packing patterns of these trienes are nearly isoenergetic. It is likely that, as for the larger π-system of DPH than that of DPB, the face-to-face stacking arrangements are considerably stabilized by CT (and π-π) interactions, but significantly destabilized by π-π repulsion [81]. Further, no photoreactivity of crystals **18** and **20** was rather unexpected, considering that diene crystals **13** and **15** were photoreactive. This may possibly be due to the difference in the magnitude of π-orbital interactions in the excited state for the diene and triene systems.

### 2.5. Halogen Substitution

#### 2.5.1. Chlorine substitution

It is well-known that chlorine substitution is effective to steer stacking arrangements suitable for [2+2] photocycloaddition of aromatic olefins [6,9,63]. The effects are mainly based on the attractive Cl···Cl interactions [83,84,85,86,87]. Typical examples include 2,4- and 2,6-dichloro substitution for (*E*)-CA [24,88], (*E*)-stilbene [89,90], (*E*,*E*)-DPB and related molecules [43,91,92,93].

**Scheme 9:**
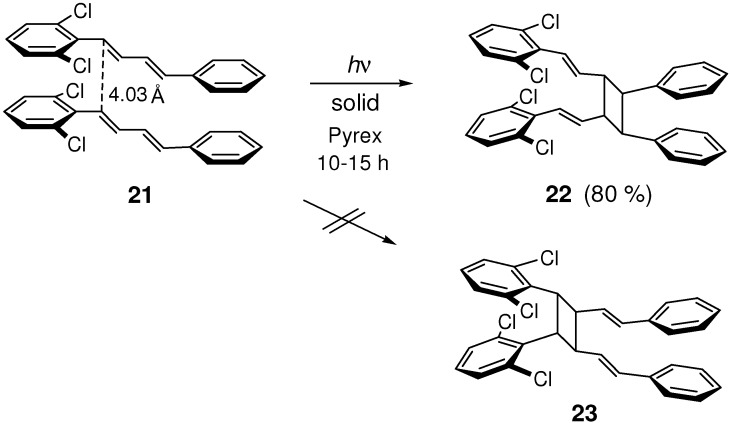
[2+2] Photodimerization of **21**.

Irradiation of 2,6-dichloro-substituted (*E*,*E*)-DPB **21** gave mirror-symmetric dimer **22** in high yield (Scheme 9). Excimer emission in the solid state was observed in this case [43,93]. Of the two possible isomers **22** and **23**, only one kind of dimer **22** was formed from **21**, which was explained in terms of twisting of 2,6-dichlorophenyl ring from the butadiene plane [43] and intramolecular conformational changes in the excimer formation [93]. 

**Scheme 10:**
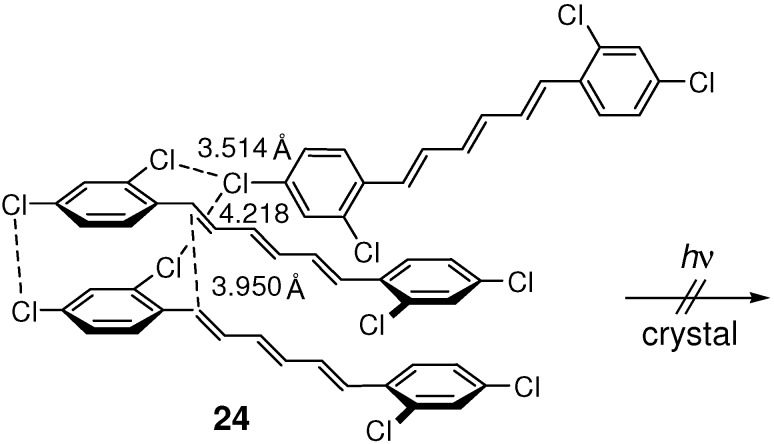
Molecular arrangements and multiple Cl···Cl interactions in photostablecrystal **24**.

In the crystal structure of 2,2',4,4'-tetrachloro-substituted (*E*,*E*,*E*)-DPH **24**, molecules were linked *via* Cl···Cl interactions with the shortest intermolecular Cl···Cl distance of 3.514 Å (Scheme 10) [94]. The molecules were further joined through π-π stacking interactions with the double bond distance of 3.950 Å. The closely stacked molecular arrangement was clearly a result of chlorine ring-substitution. Despite the relatively short distance between double bonds, however, **24** was unreactive in the solid state [45]. No observation of excimer fluorescence suggests the presence of only weak orbital-orbital interactions between the stacking molecules [59]. 

The photostability of **24** may at least partially be due to multiple Cl···Cl interactions between adjacent molecules, which will prevent easy atomic and molecular movements required by the [2+2] reaction in crystal [94].

#### 2.5.2. Fluorine substitution

It has recently been recognized that fluorine substitution [67,68,95,96] is probably more useful than chlorine substitution for constructing [2+2] photoreactive crystals [97,98,99,100,101,102,103]. In particular, noncovalent intermolecular interaction between C_6_F_5_ and C_6_H_5_ rings is known to be strongly attractive [104,105,106,107,108,109]. The interaction is widely used in crystal engineering as a strong supramolecular synthon to steer face-to-face stacking arrangements of aromatic molecules [110,111,112,113,114,115,116,117,118,119]. It can be utilized to prealign molecules not only for [2+2] photocycloaddition of olefins [120,121,122,123], but also for photo-polymerization of diacetylenes in the crystalline state [124,125].

**Scheme 11:**
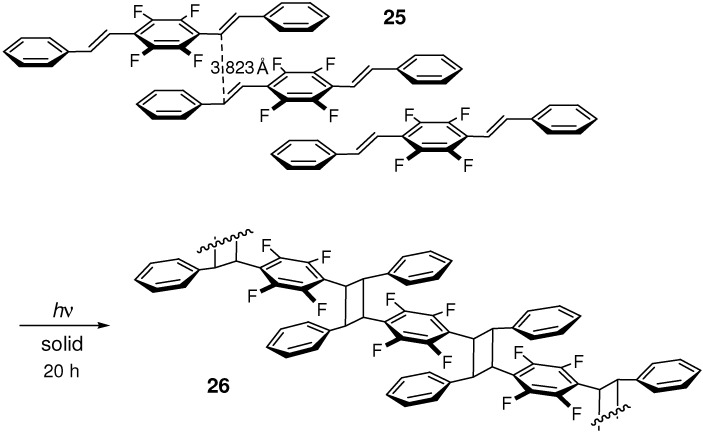
[2+2] Photodimerization and photopolymerization of **25**.

**Scheme 12:**
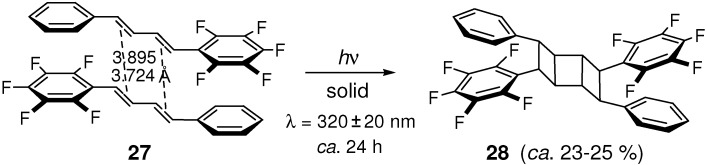
Double [2+2] photocycloaddition dimerization of **27**.

The C_6_F_5_···C_6_H_5_ interaction induced [2+2] cycloaddition of (*E*)-CA, (*E*)-stilbene, and (*E*,*E*)-distyrylbenzene molecules [120]. Irradiation of center-ring perfluorinated (*E*,*E*)-distyrylbenzene **25** yielded a white powder that was virtually insoluble in common organic solvents. The toluene-soluble part of the product was shown by GPC analysis to be an oligomeric mixture (predominantly dimer to tetramer) (**26**; the proposed structure) (Scheme 11).

(*E*,*E*)-1-Pentafluorophenyl-4-phenyl-1,3-butadiene **27** underwent double [2+2] photocycloaddition in the solid state to afford ladder-shaped dimer **28** (Scheme 12) [121]. Considering no reactivity of parent DPB, it is clear that perfluorination of the benzene ring brings remarkably the reactant molecules into head-to-tail stacking arrangement in the crystal lattice. For the centro-symmetrically related reactive double bonds, the center-to-center distances were reported to be 3.724 and 3.895 Å. The low yield of photoproduct was ascribed to the stepwise reaction mechanism for the dimerization.

**Figure 5:**
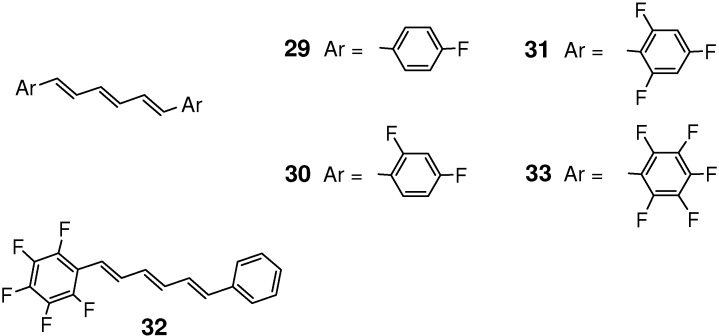
Chemical structures of fluorinated (*E*,*E*,*E*)-DPHs **29**-**33**.

For a series of ring-fluorinated (*E*,*E*,*E*)-DPHs **29**-**33** (Figure 5), the relationship between crystal structure and photophysical/photochemical properties was systematically investigated [122]. Fluorination at 4-, 2,4-, or 2,4,6-positions of the two terminal benzene rings of (*E*,*E*,*E*)-DPH was ineffective to induce [2+2] photocycloaddition. In the crystal structure, molecules **29** were arranged in a typical herringbone fashion, unfavorable for the photoreaction. Although molecules **30** and **31** were π-stacked in the lattice, the planes of the nearest two molecules were largely offset along the short molecular axes. For the nearest stacking molecules, the double bond distances [7.515 Å (=*c*) for **30** and 7.217 Å (=*b*) for **31**] were too large for the reaction to occur.

**Figure 6:**
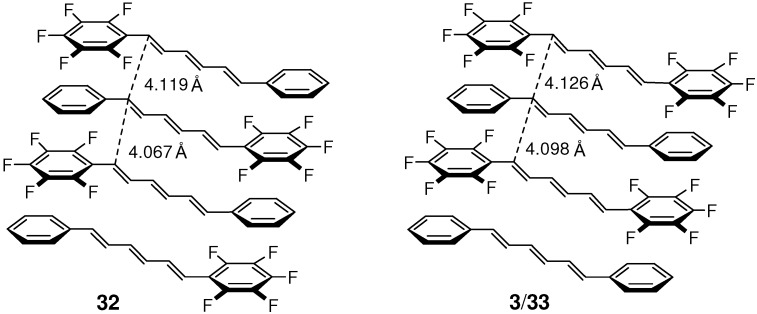
Stacking molecular arrangements in crystal **32** and **3**/**33**.

**Scheme 13:**
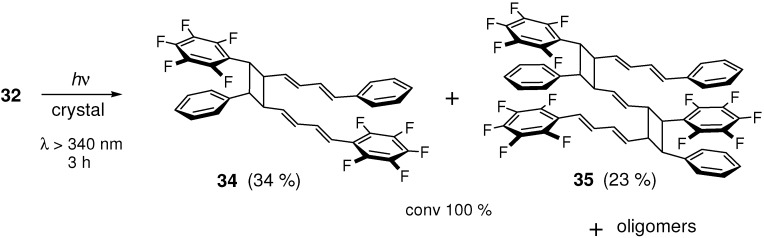
[2+2] Photodimerization and photopolymerization of **32**.

On the other hand, crystal **32** and 1:1 cocrystal **3**/**33** (Figure 6) were highly photoreactive. The [2+2] reaction of **32**, in particular, was much efficient compared to those of typical organic solids. The conversion reached 100% after only 3 h-irradiation. The photoproduct was a mixture of dimer **34**, trimers (**35**; the most plausible structure of the main trimer), and higher oligomers, all soluble in common organic solvents such as dichloromethane and acetonitrile (Scheme 13). Although the photoreaction in solution occurred inefficiently to give a mixture of several kinds of dimers, only one kind of dimer (and its mirror-image) was predominantly formed in the solid state [123]. Unlike in crystal **27** [121], the double [2+2] cycloaddition giving a ladder-shaped dimer did not take place in **32**. The photoreaction of cocrystal **3**/**33** was similar but somewhat less efficient than the reaction of **32** [123].

As a result of effective C_6_F_5_···C_6_H_5_ interactions, the molecules in crystals **32** and **3**/**33** were very similarly arranged in a π-stacking fashion (Figure 6) [122]. The double bond distances were 4.067 and 4.119 Å for **32**, and 4.098 and 4.126 Å for **3**/**33**. The reaction efficiency and the highest MW of the photoproduct for **32** and **3**/**33** were considerably enhanced when compared to those for **4** and **7**, although the double bond distances of the stacked molecules in the original structures were not greatly different for these crystals. This suggests that the C_6_F_5_···C_6_H_5_ stacking interactions are more effective than the π-π stacking interactions to keep molecules in nearly face-to-face arrangements during the [2+2] photoreaction, in which the bulky cyclobutane products will destroy the initially ordered molecular alignments.

**Figure 7:**
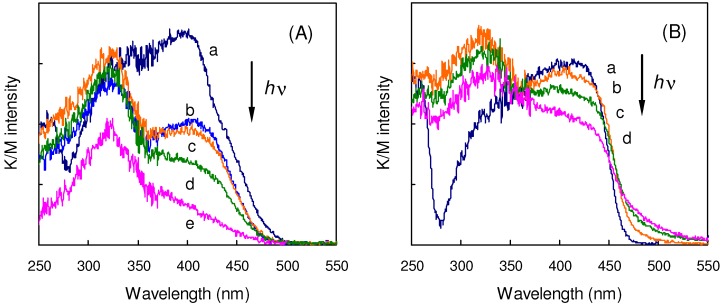
(A) Solid-state absorption spectra of **32** (a) before and after irradiation for (b) 15, (c) 30, (d) 60, and (e) 90 min;(B) Solid-state absorption spectra of **3**/**33** (a) before and after irradiation for (b) 60,(c) 120, and (d) 240 min.

Figure 7(A) shows the changes in solid-state absorption spectra of **32** obtained by Kubelka/Munk (K/M) conversion of the diffuse reflectance spectra during the photoreaction. On irradiation, the absorption band of the starting monomer around 400 nm decreased in intensity, and a new band of the dimeric and polymeric photoproducts was growing up around 325 nm. The large blue-shift of 70-80 nm in the absorption indicates the destruction of DPH π-conjugated system by the formation of aliphatic cyclobutane ring. The spectroscopic changes during the reaction of **3**/**33** were fundamentally similar (Figure 7(B)). The initially observed band around 410 nm decreased in intensity on irradiation, and the product band newly appeared around 320 nm.

**Figure 8:**
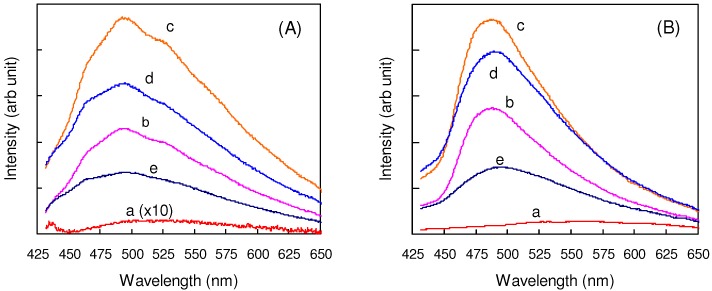
(A) Solid-state fluorescence spectra of **32** (a) before and after irradiation for (b)10, (c) 20, (d) 60, and (e) 120 min; (B) Solid-state fluorescence spectra of **3**/**33** (a) before and after irradiation for (b)10, (c) 20, (d) 60, and (e) 120 min.

Figure 8(A) shows the changes in the solid-state fluorescence spectra during the photoreaction of **32**. Before irradiation, the weak emission band due to excimeric species was observed around 525 nm, indicating strong π-orbital interactions between stacked molecules in the original crystal [122]. On irradiation, a new band centered at 495 nm was rapidly growing. This blue-shifted strong emission originated probably from unreacted monomers that were isolated in the crystal lattice with the progress of the reaction. When irradiated further, the intensity of the monomer emission gradually decreased, and finally a broad band remained only weakly around 500 nm. This suggests that the cyclobutane products are practically nonfluorescent. Similar spectral changes were observed for **3**/**33** (Figure 8(B)). 

In this case, the weak fluorescence band initially observed around 565 nm was assigned to the emission from molecular aggregates [122]. Whereas, the band around 490 nm growing on irradiation would be due to unreacted monomers isolated in the lattice. For **32** and **3**/**33**, the solid-state absorption and emission spectra thus dramatically changed before and after irradiation.

**Figure 9:**
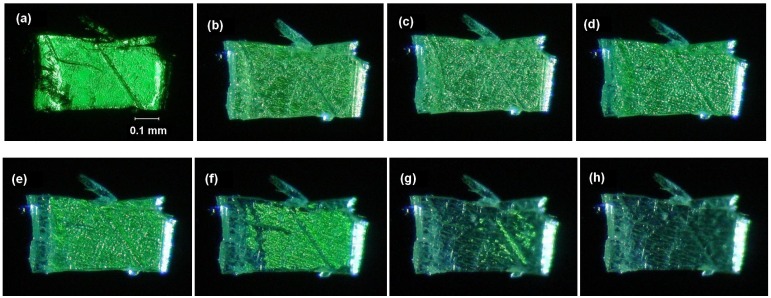
Polarizing optical micrographs of cocrystal **3/33** (a) before and after irradiation for (b) 15, (c) 30, (d) 60, (e) 120, (f) 180, (g) 240, and (h) 300 min.

Figure 9 shows the changes in the polarizing optical micrographs of cocrystal **3/33** during the photoreaction. The results can be compared with those of crystal **32** reported previously [123]. On irradiation, stripe-like microstructures formed very rapidly on the crystal surface. The formation of such microstructures was not observed for **32**. When irradiated further, the structures gradually disappeared and a transparent part was slowly growing up from the edge of the crystal. When the reaction was completed after 5 h, the crystal became almost completely transparent. This shows the photoproducts to be amorphous, in agreement with the observations in powder XRD pattern measurements [123].

**Scheme 14:**
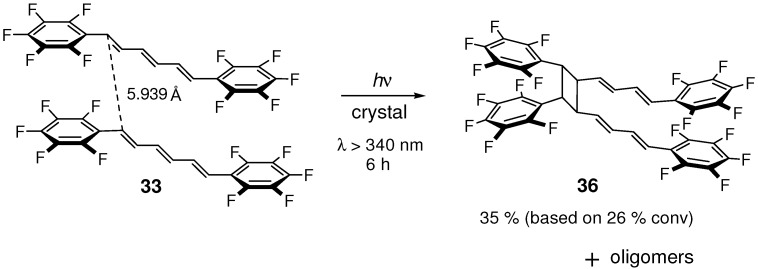
[2+2] Photodimerization and photopolymerization of **33**.

The noncovalent interaction between two C_6_F_5_ rings was also used to steer stacking arrangements [126] and to induce [2+2] photocycloaddition [127] of olefin crystals. Irradiation of **33** gave dimer **36**, formed by the reaction at the terminal double bonds of trienes, and a small amount of higher oligomers (Scheme 14) [123]. The offset for the stacking molecules in **33** was larger than those in **32** and **3**/**33**. This results from weak C_6_F_5_···C_6_F_5_ intermolecular interactions relative to C_6_F_5_···C_6_H_5_interactions [128]. For the reacting two molecules of **33** in the stack, the distance between the terminal triene carbons was 5.939 Å, considerably larger than the (normal) upper limit of 4.2 Å for the [2+2] reaction. Consistent with its photoreactivity, even from this crystal excimer fluorescence was observed with monomer emission, indicating the presence of a significant degree of intermolecular π-orbital interactions in the excited state [122].

Also, trifluoromethyl groups have been proven to be effective to steer a parallel, offset stacked orientation suitable for [2+2] cycloaddition of aromatic olefins [129,130,131].

**Scheme 15:**
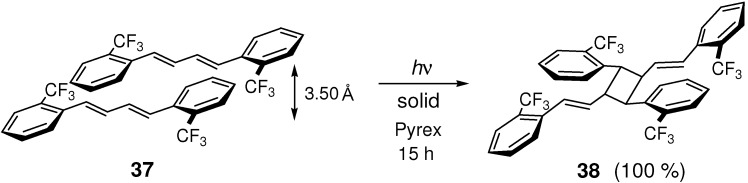
[2+2] Photodimerization of **37**.

Trifluoromethyl-substituted (*E*,*E*)-DPB **37** was irradiated to afford dimer **38** in the solid state (Scheme 15) [129]. In the crystal structure of **37**, the distance between the layers of molecule was 3.50 Å. UV-irradiation in solution resulted in the conversion of **37** to its *Z*-*E* isomer in >95% yield.

For (*E*,*E*)-1,4-bis[2,4-di(trifluoromethyl)phenyl]-1,3-butadiene, noncovalent π-π stacking interaction is considered to be mainly responsible for the observed offset stacked orientation [130]. In the crystal structure, olefins of neighboring layers were separated by 3.55 Å. UV-irradiation of the diene crystal for 15 h at room temperature yielded a [2+2] dimer as a single product in 80 % yield.

### 2.6. Substitution with Electron-Donating Groups

As described above, ring-substitution of (*E*,*E*,*E*)-DPH with electron-withdrawing cyano, formyl groups, or fluorine atoms induced solid-state [2+2] photocycloaddition at the triene double bonds. Up to now, however, no examples have been found for the photoreactive DPHs having electron-donating substituents. Thus, 4,4'-bis(*N*,*N*-dimethylamino)-substituted (*E*,*E*,*E*)-DPH and 4,4'-dimethoxy-substituted (*E*,*E*,*E*)-DPH were photostable in the solid state [45].

Although the single-crystal X-ray structure analysis of 4,4'-dimethoxy DPH has been unsuccessful at present, the two-dimensional ^1^H spin-exchange NMR spectrum showed that the methoxy group was located close to the triene chain of an adjacent molecule in the solid state [132]. This suggests that the molecular planes are stacked with a large offset along the long molecular axis. Therefore, the photostability of the 4,4'-dimethoxy derivative may at least partially be due to molecular alignment unfavorable for the [2+2] reaction. Further, since H atoms of the methoxy group are 'out-of-plane' atoms, this may possibly lead to the interplanar distance larger than 4 Å [63].

## 3. Atomic and Molecular Movements in Crystals during [2+2] Photodimerization and Photopolymerization

According to Schmidt's rule, the distances between the potentially reactive double bonds for topochemical [2+2] photocycloaddition should be less than *ca.* 4.2 Å [1,2,25]. However, several or more exceptions have been reported to this rule up to now [7,9]. Among the DPH derivatives described above, for example, **9**, **20** and **24** were unreactive, although the double bond distances were shorter than 4.2 Å in the crystal structures. On the other hand, **33** was reactive, although the double bond distance of 5.939 Å was much longer than the criterion of 4.2 Å (Table 1).

**Table 1:** Double bond distance and [2+2] photoreactivity for (*E*,*E*,*E*)-DPH crystals.

Crystal	Double bond distance *^a^* (Å)	[2+2] Photoreactivity
**3**	7.730	no
**4**	3.928	yes
**7**	3.926	yes
**9**	3.871	no
**20**	3.862	no
**24**	3.950	no
**32**	4.067, 4.119	yes
**3/33**	4.098, 4.126	yes
**33**	5.939	yes

*^a^* distance between the terminal triene carbons for the nearest stacking molecules.

For [2+2] cycloaddition to occur, atomic and molecular movements required by the formation of cyclobutane ring should be possible. When the interactions between the adjacent molecules are too strong, the movements will become difficult or almost impossible [133,134,135]. In this context, we can say that intermolecular interactions, used for constructing the stacking structure favorable for the [2+2] reaction, should be rather *weak* than too strong for the reaction to occur. Coumarin-3-carboxylic acid, for example, was photoreactive whereas 5-bromouracil and maleic acid were photostable, although the double bond distances were less than 4.2 Å in all three molecules [134]. The photostability of5-bromouracil and maleic acid was attributed to the presence of a much more extensive hydrogen bonding network in these two structures than in coumarin-3-carboxylic acid. Also for 1,3-phenylene-diacrylic acid, only the olefinic bonds adjacent to carboxylic groups involved in dimeric hydrogen bonds were photoreactive, while those associated with polymeric hydrogen bonds were photochemically inert [135].

**Figure 10:**
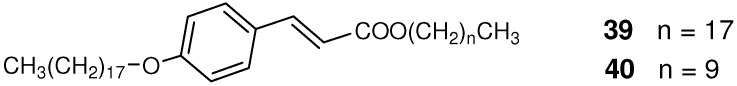
Chemical structures of cinnamates **39** and **40**.

Further, an enough space for the atomic and molecular movements in the crystal lattice is required by the reaction. For the [2+2] photodimerization of cinnamates **39** and **40** (Figure 10) in monolayers at the liquid/graphite interface, neither of the two molecules exhibited monomer packing ideal for the [2+2] reaction [136]. The double bonds were both separated by the distances larger than 4.2 Å. However, molecules **39** in monolayer, having a less closely packed adlayer structure, were photodimerizable. The photoreactivity was explained by means of packing fluctuations, allowing the reactive centers approach each other. While molecules **40**, having an interdigitated structure, were resistant to photodimerization. In addition to the increased intermolecular distance, the interdigitation of the monomer molecules limited packing fluctuations. These were clearly evidenced by direct observation of the photoreaction using STM. The importance of a free space for molecular movements (conformational change) in the crystal lattice has also been pointed out for the photodimerization of two kinds of bulky olefins with 1,4-dihydropyridine skeleton [137].

Since [2+2] cycloaddition is a photoreaction of olefins, it requires sufficient interaction between π-orbitals of double bonds of the reacting two (or more) molecules, at least one of which is in the electronic excited state. Therefore, even if the double bond distance between the ground-state molecules determined by X-ray structure analysis is shorter than 4.2 Å, the reaction will not occur when the π-orbital interaction in the excited state is insufficient [133,138,139,140,141]. On the contrary, even if the double bond distance in the crystal structure is somewhat longer than 4.2 Å, the reaction can take place when the orbital interaction is enough [72,141,142]. Thus, the magnitude of orbital-orbital interaction in the excited state should be more important than the X-ray distance between double bonds in determining the [2+2] photoreactivity.

On the other hand, there are a few examples of [2+2] photoreactive olefin crystals in which double bond distances are much larger than 4.2 Å. Crystal **33** is one of such examples as described above. For [2+2] photodimerization in a self-assembled monolayer of 4-amyloxy CA on Au (111), direct observation using STM showed that the atoms and molecules moved in the structure during the photoreaction [143]. The molecular distance in the original monolayer was 6.0 Å. In this case, it is considered that photoexcitation creates short-term lattice instability [71,144,145,146], which drives one molecule close to a neighbor and gives the molecule a more favorable orientation so as to cause a photoreaction [143]. In the excited state, molecules are expected to be more attractive each other than in the ground state. Also for the [2+2] dimerization of 2-benzyl-5-benzilidenecyclopentanone derivatives, monitoring structural transformations in crystals using single-crystal XRD revealed that the reactant molecules were moving and even approaching each other during the photoreaction [147,148,149]. Such large molecular movements during the solid-state [2+2] reactions of olefins have also been suggested by other experimental observations [150,151,152]. 

## 4. Industrial Applications

### 4.1. Amorphous Materials

**Figure 11:**
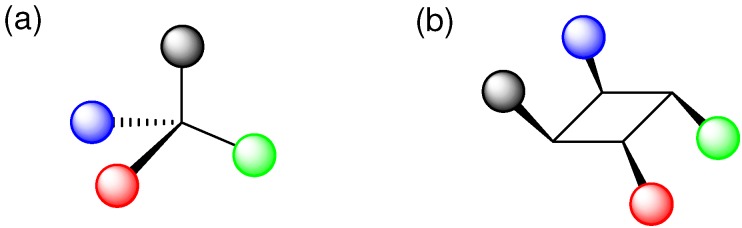
Four functional groups in (a) tetrahedral and (b)cyclobutane molecules.

For amorphous molecular materials, very disordered structures are usually required [153,154]. [2+2] Photocycloaddition of olefins will easily give highly disordered and bulky structures. As in a molecule having a tetrahedral carbon framework [153], various functional groups can be attached as four arms to the cyclobutane ring carbons, leading to the formation of 'functional amorphous materials' (Figure 11).

The [2+2] photoproducts from crystals **32** and **3**/**33** were amorphous as shown above [123]. The [2+2] reaction of 1,3-phenylenediacrylic acid dimethyl ester [155,156] and α,α'-dicyano-1,4-phenylenediacrylic acid dimethyl ester [157] also gave amorphous oligomers.

### 4.2. Photocrosslinking Materials

**Scheme 16:**
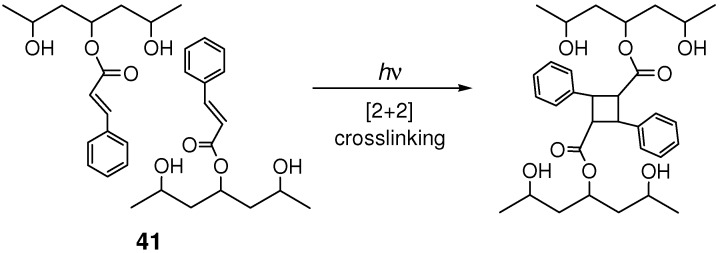
Photocrosslinking reaction of poly(vinyl cinnamate) **41**.

Poly(vinyl cinnamate) **41** would be one of the most important photosensitive polymers. UV-light irradiation of the polymer gives a material with reduced solubility, which is considered to be due to photocrosslinking between cinnamoyl pendant groups in different polymer chains. The mechanism of photocrosslinking is proposed to be [2+2] cycloaddition of the olefinic groups (Scheme 16) [158,159,160,161,162,163,164].

**Figure 12:**
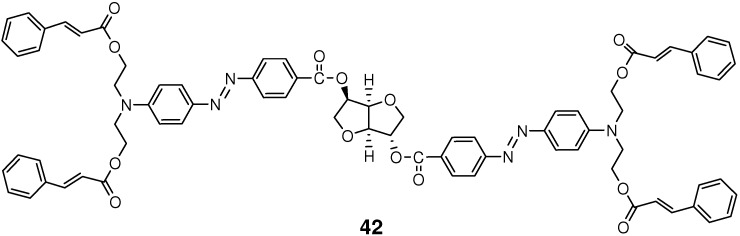
Chemical structure of azo monomer **42**.

Amorphous azo monomer **42** (Figure 12) having four cinnamate arms underwent [2+2] cycloaddition upon UV-light irradiation [165]. The monomer readily formed surface relief structures upon Ar^+^ laser irradiation, and the resulting structures were further stabilized through a photocrosslinking reaction induced by UV-light irradiation. On the basis of the material, two-dimensional quasi-crystal structures with different rotation symmetries were fabricated by using the dual-beam multiple exposure technique. The quasi-crystal structures were prepared simply through multistep light irradiation, and no subsequent wet-etch or dry-etch step was required in the process.

### 4.3. Photochemical Crack Healing in Polymeric Materials

**Scheme 17:**
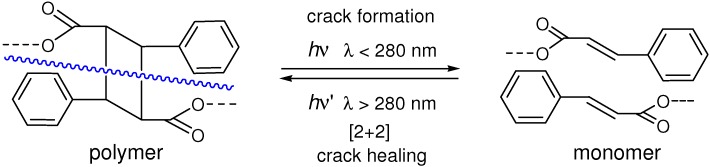
Photochemical crack healing *via* [2+2] cycloaddition.

[2+2] Photocycloaddition of cinnamoyl groups can be used as a crack healing reaction in polymeric materials [166]. A photocrosslinkable cinnamate monomer, 1,1,1-tris(cinnamoyloxymethyl)ethane, was irradiated with UV-light (λ > 280 nm) to give a transparent, insoluble film by crosslinking *via* the [2+2] reaction. It was expected that cyclobutane would reverse to original cinnamoyl structure upon crack formation and propagation, and the crack healing could be accomplished by the re-cycloaddition of cinnamoyl groups (Scheme 17). The photochemical healing proceeded very fast and did not require any catalyst, additive or severe heat-treatment.

### 4.4. Optical Memories and Fluorescence Switches

In the [2+2] photocycloaddition of highly conjugated molecules, the delocalized π-electron systems of olefin monomers are broken by the formation of aliphatic cyclobutane rings of dimeric or polymeric products. The solid-state fluorescence spectra, in general, dramatically change before and after the photoreaction, as the examples are shown above for **32** and **3**/**33** (Figure 8). Therefore, if the [2+2] reactions are photochemically reversible, they can be used for optical memories and fluorescence switches. 

**Scheme 18:**
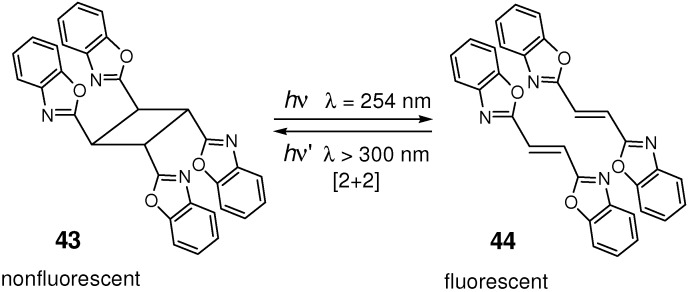
Photocleavage of **43** and re-cycloaddition of **44**.

For tetra(2-benzoxazolyl)cyclobutane **43**, the four benzoxazoles were electronically isolated and exhibited no fluorescence emission [167]. On irradiation with UV-light (λ = 254 nm), **43** cleaved to 1,2-di(2-benzoxazolyl)ethene **44**, which showed strong emission at 420 nm. The reverse reaction occurred on UV-light (λ > 300 nm) irradiation (Scheme 18). Thus, **43** and **44** show typically optical bistability as a fluorescent switch, which can be applied in thermally stable, rewritable optical data storage. Most importantly, the fluorescence emission properties of conjugated molecules are able to be controlled only by irradiation of light.

**Scheme 19:**
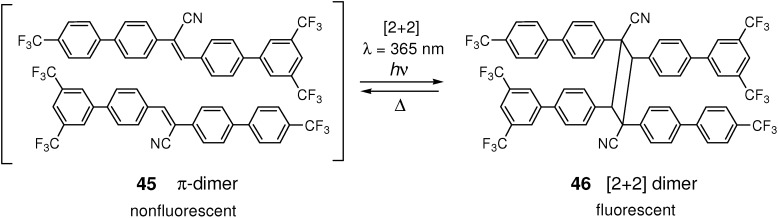
[2+2] Photocycloaddition of **45** and the reverse reaction from **46**.

In contrast, the crystal of cyano-stilbene derivative having trifluoromethyl substituents **45** was nonfluorescent due to the formation of π-dimer system but switched to highly fluorescent **46** when an external shear-strain and/or prolonged UV-light (λ = 365 nm) irradiation was applied (Scheme 19) [168]. The fluorescence modulation is due to the external and/or internal shear-induced lateral displacement of the π-dimer molecular pair. The fluorescence emission occurred at the cost of frustrated [2+2] cycloaddition. In this case the reverse reaction from **46** took place thermally. Thus the system provides an example of reversible fluorescence switching in the solid state. 

## 5. Conclusions

Although having multiple reactive double bonds, the unsubstituted α,ω-diphenylpolyenes, (*E*,*E*)-DPB and (*E*,*E*,*E*)-DPH, underwent no [2+2] photocycloaddition in the solid state due to the unfavorable crystal packing for the reaction. However, with appropriate ring-substituents, noncovalent intermolecular interactions in crystals were effectively utilized to prealign molecules in stacking arrangements, suitable for the [2+2] photodimerization and photopolymerization.

Cyano- and formyl-substituted DPHs, **4** and **7** respectively, underwent solid-state [2+2] cycloaddition to give dimers and higher oligomers. The crystal structures were constructed by relatively weak intermolecular interactions such as CH···N and CH···O hydrogen bonds. It seems to be important that the interactions in the [2+2] photoreactive crystals should be rather weak than too strong, so as to make atomic and molecular movements required by the reaction easy in the crystal lattice.

The [2+2] photoreaction of perfluorinated DPHs **32** and **3**/**33** occurred more efficiently than those of **4** and **7** to afford dimers, trimers, and higher oligomers in moderate to reasonably good yields. The novel structures of the photoproducts were almost inaccessible by other synthetic methods. However, MWs of the polymeric products were relatively low. To improve this point, the positions of reactant molecules in crystals should not change largely during the photoreaction. For this purpose, it would be desirable to perform the reaction in a single-crystal-to-single-crystal manner, by controlling the light energy of irradiation (irradiation at the long wavelength tail of the absorption) [27,169,170] or by nanocrystallization of the reactants [171,172].

Interestingly, symmetrically perfluorinated DPH **33** was [2+2] photoreactive, despite the large distance of double bonds (5.939 Å) in the crystal. The result suggests that molecules can move and approach each other in the lattice on photoexcitation.

The chloro and donor-acceptor substituents were useful as steering groups for the [2+2] reactions of (*E*)-CA, and those of shorter α,ω-diphenylpolyenes such as (*E*)-stilbene and (*E*,*E*)-DPB. However, the substitution was ineffective to induce the reactions of (*E*,*E*,*E*)-DPHs. Despite the π-stacked molecular arrangements with double bond distances less than 4.2 Å, crystals **9**, **20** and **24** were photostable. Although the reasons are unclear at present, the results suggest that the magnitude of π-orbital interactions in the excited state is more important in determining the [2+2] photoreactivity than the X-ray double bond distance in the ground state. This is consistent with the fact that the observation of solid-state excimer/monomer fluorescence correlates very well with [2+2] photoreactivity/unreactivity for all the DPH crystals examined.

The solid-state [2+2] photocycloaddition is one of the most useful photochemical reactions, not only in synthetic organic chemistry but also in material chemistry and applied physics. Only by light irradiation, very large changes in molecular structure are induced, which should consequently lead to large changes in electronic and optical properties of the molecules. Thus, if photochemical and/or thermal reverse reactions are available, the [2+2] reactions will find a wide variety of industrial applications.

## References

[1] Schmidt G.M.J. (1971). Photodimerization in the solid state. Pure Appl. Chem..

[2] Cohen M.D. (1975). The photochemistry of organic solids. Angew. Chem. Int. Ed. Engl..

[3] Green B.S., Lahav M., Rabinovich D. (1979). Asymmetric synthesis *via* reactions in chiral crystals. Acc. Chem. Res..

[4] Gavezzotti A., Simonetta M. (1982). Crystal chemistry in organic solids. Chem. Rev..

[5] Ramamurthy V. (1986). Organic photochemistry in organized media. Tetrahedron.

[6] Ramamurthy V., Venkatesan K. (1987). Photochemical reactions of organic crystals. Chem. Rev..

[7] Murthy G.S., Arjunan P., Venkatesan K., Ramamurthy V. (1987). Consequences of lattice relaxability in solid state photodimerizations. Tetrahedron.

[8] Ohashi Y. (1988). Dynamical structure analysis of crystalline-state racemization. Acc. Chem. Res..

[9] Ramamurthy V. (1991). Photochemistry in Organized and Constrained Media.

[10] Koshima H., Matsuura T. (1996). Solid state photoreactions occurring at the interface between crystallites of two different organic compounds. J. Photochem. Photobiol. A Chem..

[11] Ito Y. (1998). Solid-state photoreactions in two-component crystals. Synthesis.

[12] Sonoda Y., Horspool W., Lenci F. (2004). [2+2]-Photocycloadditions in the solid state. CRC Handbook of Organic Photochemistry and Photobiology.

[13] Friščić T., MacGillivray L.R. (2005). Single-crystal-to-single-crystal [2+2] photodimerizations: From discovery to design. Z. Kristallogr..

[14] Toda F. (1995). Solid state organic chemistry: Efficient reactions, remarkable yields, and stereoselectivity. Acc. Chem. Res..

[15] Tanaka K., Toda F. (2000). Solvent-free organic synthesis. Chem. Rev..

[16] Toda F. (2005). Thermal and photochemical reactions in the solid state. Top. Curr. Chem..

[17] Kaupp G. (2008). Waste-free synthesis and production all across chemistry with the benefit of self-assembled crystal packings. J. Phys. Org. Chem..

[18] Maekawa Y., Lim P.-J., Saigo K., Hasegawa M. (1991). Preparation of a crystalline linear high copolymer by topochemical photopolymerization of diolefin mixed crystals. Macromolecules.

[19] Braga D., Grepioni F. (2004). Reactions between or within molecular crystals. Angew. Chem. Int. Ed..

[20] Avendaño C., Briceño A. (2009). Concomitant [2+2] cycloaddition solid state reactions from co-crystals self-assembled *via* mechanochemistry. CrystEngCommunity.

[21] Nagarathinam M., Vittal J.J. (2010). Solid-state synthesis of coordination polymers for [2+2] photoreactions by grinding. Aust. J. Chem..

[22] Peedikakkal A.M.P., Vittal J.J. (2010). Solid-state photochemical behavior of a triple-stranded ladder coordination polymer. Inorg. Chem..

[23] Cohen M.D., Schmidt G.M.J. (1964). Topochemistry. Part I. A survey. J. Chem. Soc..

[24] Cohen M.D., Schmidt G.M.J., Sonntag F.I. (1964). Topochemistry. Part II. The photochemistry of *trans*-cinnamic acids. J. Chem. Soc..

[25] Schmidt G.M.J. (1964). Topochemistry. Part III. The crystal chemistry of some *trans*-cinnamic acids. J. Chem. Soc..

[26] Quina F.H., Whitten D.G. (1977). Photochemical reactions in organized monolayer assemblies. 4. Photodimerization, photoisomerization, and excimer formation with surfactant olefins and dienes in monolayer assemblies, crystals, and micelles. J. Am. Chem. Soc..

[27] Novak K., Enkelmann V., Wegner G., Wagener K.B. (1993). Crystallographic study of a single crystal to single crystal photodimerization and its thermal reverse reaction. Angew. Chem. Int. Ed. Engl..

[28] Kole G.K., Tan G.K., Vittal J.J. (2010). Anion-controlled stereoselective synthesis of cyclobutane derivatives by solid-state [2+2] cycloaddition reaction of the salts of *trans*-3-(4-pyridyl) acrylic acid. Org. Lett..

[29] Vedernikov A.I., Kuz´mina L.G., Sazonov S.K., Lobova N.A., Loginov P.S., Churakov A.V., Strelenko Y.A., Howard J.A.K., Alfimov M.V., Gromov S.P. (2007). Styryl dyes. Synthesis and study of the solid-state [2+2] autophotocycloaddition by NMR spectroscopy and X-ray diffraction. Russ. Chem. Bull. Int. Ed..

[30] Kuz’mina L.G., Vedernikov A.I., Sazonov S.K., Lobova N.A., Loginov P.S., Howard J.A.K., Alfimov M.V., Gromov S.P. (2008). Specific features of the crystal packing that enable styryl dyes of the pyridine series to undergo the solid-phase [2+2] photocycloaddition including the process with single crystal retention. Crystallogr. Rep..

[31] Tulyakova E.V., Fedorova O.A., Fedorov Y.V., Anisimov A.V. (2008). [2+2]-Photocycloaddition reaction of self-assembled crown-containing 2-styrylpyridinium perchlorate in a solid state. J. Photochem. Photobiol. A Chem..

[32] Kuz´mina L.G., Vedernikov A.I., Lobova N.A., Sazonov S.K., Basok S.S., Howard J.A.K., Gromov S.P. (2009). Design of crystal packings of styrylheterocycles and [2+2] photocycloaddition reactions in their single crystals 6. Synthesis and crystal packings of neutral crown-containing and model styrylheterocycles. Russ. Chem. Bull. Int. Ed..

[33] Friščić T., MacGillivray L.R. (2005). Cyclophanes and ladderanes: Molecular targets for supramolecular chemists. Supramol. Chem..

[34] MacGillivray L.R., Papaefstathiou G.S., Friščić T., Hamilton T.D., Bučar D.-K., Chu Q., Varshney D.B., Georgiev I.G. (2008). Supramolecular control of reactivity in the solid state: From templates to ladderanes to metal-organic frameworks. Acc. Chem. Res..

[35] MacGillivray L.R. (2008). Organic synthesis in the solid state via hydrogen-bond-driven self-assembly. J. Org. Chem..

[36] Nagarathinam M., Vittal J.J. (2006). A rational approach to crosslinking of coordination polymers using the photochemical [2+2] cycloaddition reaction. Macromol. Rapid Commun..

[37] Nagarathinam M., Peedikakkal A.M.P., Vittal J.J. (2008). Stacking of double bonds for photochemical [2+2] cycloaddition reactions in the solid state. Chem. Commun..

[38] Han Y.-F., Lin Y.-J., Jia W.-G., Wang G.-L., Jin G.-X. (2008). Template-controlled topochemical photodimerization based on ''organometallic macrocycles'' through single-crystal to single-crystal transformation. Chem. Commun..

[39] Han Y.-F., Jia W.-G., Yu W.-B., Jin G.-X. (2009). Stepwise formation of organometallic macrocycles, prisms and boxes from Ir, Rh and Ru-based half-sandwich units. Chem. Soc. Rev..

[40] Zhang W.-Z., Han Y.-F., Lin Y.-J., Jin G.-X. (2010). [2+2] Photodimerization in the solid state aided by molecular templates of rectangular macrocycles bearing oxamidato ligands. Organometallics.

[41] Hasegawa M. (1983). Photopolymerization of diolefin crystals. Chem. Rev..

[42] Hasegawa M. (1995). Photodimerization and photopolymerization of diolefin crystals. Adv. Phys. Org. Chem..

[43] Cohen M.D., Elgavi A., Green B.S., Ludmer Z., Schmidt G.M.J. (1972). Photodimerization and excimer emission in a crystalline 1,4-diphenylbutadiene. J. Am. Chem. Soc..

[44] Singh A.K., Krishna T.S.R. (1997). Fluorescence and photodimerization studies of cyano-substituted diphenylbutadienes. J. Phys. Chem. A.

[45] Sonoda Y., Miyazawa A., Hayashi S., Sakuragi M. (2001). Intermolecular [2+2] photocycloaddition of formyl- and cyano-substituted diphenylhexatrienes in the solid state. Chem. Lett..

[46] Drenth W., Wiebenga E.H. (1953). Structure of α,ω-diphenylpolyenes. 1. Crystal data of 1,4-diphenyl-1,3-butadiene, 1,6-diphenyl-1,3,5-hexatriene and 1,8-diphenyl-1,3,5,7-octatetraene. Recl. Trav. Chim. Pays-Bas.

[47] Glaser R., Dendi L.R., Knotts N., Barnes C.L. (2003). Ab initio and crystal structures of (*E*,*E*)-1,4-diphenylbutadiene: A new type ofarene-arene double T-contact and an interesting interlayer cooperation involving diastereoisomeric contacts. Cryst. Growth Des..

[48] Gavezzotti A., Desiraju G.R. (1988). A systematic analysis of packing energies and other packing parameters for fused-ring aromatic hydrocarbons. Acta Crystallogr..

[49] Desiraju G.R., Gavezzotti A. (1989). Crystal structures of polynuclear aromatic hydrocarbons. Classification, rationalization and prediction from molecular structure. Acta Crystallogr..

[50] Hall T., Bachrach S.M., Spangler C.W., Sapochak L.S., Lin C.T., Guan H.W., Rogers R.D. (1989). Structure of all-*trans*-1,6-diphenyl- (*A*) and all-*trans*-1,6-bis(*o*-methoxyphenyl)-1,3,5-hexatriene (*B*). Acta Crystallogr..

[51] Sonoda Y., Morii H., Sakuragi M., Suzuki Y. (1998). Substituent effect on the *cis-trans* photoisomerization of *trans,trans,trans*-1,6-diphenyl-1,3,5-hexatrienes. Chem. Lett..

[52] Sonoda Y., Kwok W.M., Petrasek Z., Ostler R., Matousek P., Towrie M., Parker A.W., Phillips D. (2001). Solvent effects on the photophysical and photochemical properties of (*E*,*E*,*E*)-1,6-bis(4-nitrophenyl)hexa-1,3,5-triene. J. Chem. Soc. Perkin Trans. 2.

[B53-molecules-16-00119] 53.The crystal structures of **4** and **7** are to be published elsewhere

[54] Reddy D.S., Goud B.S., Panneerselvam K., Desiraju G.R. (1993). C–H···N mediated hexagonal network in the crystal structure of the 1:1 molecular complex 1,3,5-tricyanobenzene-hexamethylbenzene. J. Chem. Soc. Chem. Commun..

[55] Desiraju G.R. (1995). Supramolecular synthons in crystal engineering—A new organic synthesis. Angew. Chem. Int. Ed. Engl..

[56] Desiraju G.R. (1997). Designer crystals: Intermolecular interactions, network structures and supramolecular synthons. Chem. Commun..

[57] Nangia A., Desiraju G.R. (1998). Supramolecular structures - reason and imagination. Acta Crystallogr..

[58] Dhurjati M.S.K., Sarma J.A.R.P., Desiraju G.R. (1991). Unusual [2+2] topochemical cycloadditions of 3-cyano- and 4-cyano-cinnamic acids: Temperature dependent solid state photochemical reactions. J. Chem. Soc. Chem. Commun..

[59] Sonoda Y., Kawanishi Y., Ikeda T., Goto M., Hayashi S., Yoshida Y., Tanigaki N., Yase K. (2003). Fluorescence spectra for the microcrystals and thin films of *trans,trans,trans*-1,6-diphenyl-1,3,5-hexatrienes. J. Phys. Chem. B.

[60] Sonoda Y., Suzuki Y. (1996). Stereoselective *Z*,*E*-photoisomerization of formyl-substituted (*E*,*E*,*E*)-1,6-diphenylhexa-1,3,5-triene in solution. J. Chem. Soc. Perkin Trans. 2.

[61] Sonoda Y., Suzuki Y. (1996). Solvent-dependent *cis-trans* one-way photoisomerization of bisformyl-substituted 1,6-diphenyl-1,3,5-hexatriene. Chem. Lett..

[62] Sonoda Y., Kawanishi Y., Sakuragi M. (1999). A heavy-atom effect on the *cis*-*trans* photoisomerization of bisforlmyl-substituted *trans,trans,trans*-1,6-diphenyl-1,3,5-hexatriene. Chem. Lett..

[63] Sarma J.A.R.P., Desiraju G.R. (1986). The role of Cl···Cl and C–H···O interactions in the crystal engineering of 4-A short-axis structures. Acc. Chem. Res..

[64] Desiraju G.R. (1991). The C–H···O hydrogen bond in crystals: What is it?. Acc. Chem. Res..

[65] Desiraju G.R. (1996). The C–H···O hydrogen bond: Structural implications and supramolecular design. Acc. Chem. Res..

[66] Steiner T., Desiraju G.R. (1998). Distinction between the weak hydrogen bond and the van der Waals interaction. Chem. Commun..

[67] Desiraju G.R. (2002). Hydrogen bridges in crystal engineering: Interactions without borders. Acc. Chem. Res..

[68] Desiraju G.R. (2005). C–H···O and other weak hydrogen bonds. From crystal engineering to virtual screening. Chem. Commun..

[69] Nakanishi F., Nakanishi H., Tasai T., Suzuki Y., Hasegawa M. (1974). Water participation in the crystalline state photoreaction photodimerization of p-formylcinnamic acid. Chem. Lett..

[70] Nakanishi F., Nakanishi H., Tsuchiya M., Hasegawa M. (1976). Water-participation in the crystalline-state photodimerization of cinnamic acid derivatives. A new type of organic photoreaction. Bull. Chem. Soc. Jpn..

[71] Ghosh U., Misra T.N. (1985). Spectroscopic studies of photochemical reactions in organic solids. Photodimerization of *p*-formylcinnamic acid. Bull. Chem. Soc. Jpn..

[72] Nakanishi H., Hasegawa M., Mori T. (1985). Structure of the β-form of *p*-formylcinnamic acid, C_10_H_8_O_3_, a photodimerizable crysta. Acta Crystallogr..

[73] Sharma C.V.K., Desiraju G.R. (1994). C–H···O hydrogen bond patterns in crystalline nitro compounds: Studies in solid-state molecular recognition. J. Chem. Soc. Perkin Trans. 2.

[74] Thaimattam R., Xue F., Sarma J.A.R.P., Mak T.C.W., Desiraju G.R. (2001). Inclusion compounds of tetrakis(4-nitrophenyl)methane: C–H···O networks, pseudopolymorphism, and structural transformations. J. Am. Chem. Soc..

[75] Sonoda Y., Kawanishi Y., Goto M. (2005). (*E*,*E*,*E*)-1,6-Bis(4-nitrophenyl)hexa-1,3,5-triene. Acta Crystallogr..

[76] Sonoda Y., Tsuzuki S., Goto M., Tohnai N., Yoshida M. (2010). Fluorescence spectroscopic properties of nitro-substituted diphenylpolyenes: Effects of intramolecular planarization and intermolecular interactions in crystals. J. Phys. Chem. A.

[77] Desiraju G.R., Sharma C.V.K.M. (1991). C–H···O hydrogen bonding and topochemistry in crystalline 3,5-dinitrocinnamic acid and its 1:1 donor–acceptor complex with 2,5-dimethoxycinnamic acid. J. Chem. Soc. Chem. Commun..

[78] Sharma C.V.K., Panneerselvam K., Shimoni L., Katz H., Carrell H.L., Desiraju G.R. (1994). 3-(3',5'-Dinitrophenyl)-4-(2',5'-dimethoxyphenyl)cyclobutane-1,2-dicarboxylic acid: Engineered topochemical synthesis and molecular and supramolecular properties. Chem. Mater..

[79] Capparelli M.V., Codding P.W. (1993). Photodimerization of α,ω-diarylbutadienes. I. Crystal and molecular structures of *trans*,*trans*-l-(2'-methoxyphenyl)-4-(4'-nitrophenyl)-l,3-butadiene and its photodimer. Can. J. Chem..

[80] Mascitti V., Corey E.J. (2006). Photochemical studies on ladderane formation from conjugated esters in solution or solid phase. Tetrahedron Lett..

[81] Sonoda Y., Goto M., Tsuzuki S., Tamaoki N. (2006). Fluorescence spectroscopic properties and crystal structure of a series of donor-acceptor diphenylpolyenes. J. Phys. Chem. A.

[82] Sonoda Y., Kawanishi Y. (2003). Solvent-dependent *cis*-*trans* photoisomerization of *p*-methoxy-*p*′-nitro-substituted *trans*,*trans*,*trans*-1,6-diphenyl-1,3,5-hexatriene. Chem. Lett..

[83] Sarma J.A.R.P., Desiraju G.R. (1985). The chloro-substituent as a steering group: A comparative study of non-bonded interactions and hydrogen bonding in crystalline chloro-aromatics. Chem. Phys. Lett..

[84] Desiraju G.R., Parthasarathy R. (1989). The nature of halogen···halogen interactions: Are short halogen contacts due to specific attractive forces or due to close packing of nonspherical atoms?. J. Am. Chem. Soc..

[85] Pedireddi V.R., Reddy D.S., Goud B.S., Craig D.C., Rae A.D., Desiraju G.R. (1994). The nature of halogen···halogen interactions and the crystal structure of 1,3,5,7-tetraiodoadamantane. J. Chem. Soc. Perkin Trans. 2.

[86] Reddy C.M., Kirchner M.T., Gundakaram R.C., Padmanabhan K.A., Desiraju G.R. (2006). Isostructurality, polymorphism and mechanical properties of some hexahalogenated benzenes: The nature of halogen···halogen interactions. Chem. Eur. J..

[87] Bui T.T.T., Dahaoui S., Lecomte C., Desiraju G.R., Espinosa E. (2009). The nature ofhalogen···halogen interactions: A model derived from experimental charge-density analysis. Angew. Chem. Int. Ed..

[88] Sarma J.A.R.P., Desiraju G.R. (1984). Crystal engineering *via* Cl···Cl non-bonded interactions. The novel 2:1 complex, 6-chloro-3,4-(methylenedioxy)cinnamic acid-2,4-dichlorocinnamic acid. Topochemical conversion into an unsymmetrical cyclobutane and kinetics of the reaction. J. Chem. Soc. Chem. Commun..

[89] Cohen M.D., Green B.S., Ludmer Z., Schmidt G.M.J. (1970). Excimer emission and photodimerization in a crystalline stilbene. Chem. Phys. Lett..

[90] Cohen R., Ludmer Z., Yakhot V. (1975). Structural influence on the excimer emission from a dimorphic crystalline stilbene. Chem. Phys. Lett..

[91] Elgavi A., Green B.S., Schmidt G.M.J. (1973). Reactions in chiral crystals. Optically active heterophotodimer formation from chiral single crystals. J. Am. Chem. Soc..

[92] Green B.S., Heller L. (1974). Solution and solid-state photodimerization of some styrylthiophenes. J. Org. Chem..

[93] Warshel A., Shakked Z. (1975). Theoretical study of excimers in crystals of flexible conjugated molecules. Excimer formation and photodimerization in crystalline 1,4-diphenylbutadiene. J. Am. Chem. Soc..

[94] Sonoda Y., Kawanishi Y., Goto M. (2003). (*E*,*E*,*E*)-1,6-Bis(2,4-di-chloro-phenyl)-hexa-1,3,5-triene. Acta Crystallogr..

[95] Thalladi V.R., Weiss H.-C., Bläser D., Boese R., Nangia A., Desiraju G.R. (1998). C–H···F interactions in the crystal structures of some fluorobenzenes. J. Am. Chem. Soc..

[96] Desiraju G.R. (2007). Crystal engineering: A holistic view. Angew. Chem. Int. Ed..

[97] Kumar V.A., Begum N.S., Venkatesan K. (1993). Crystal engineering: Fluorine as a new steering group. J. Chem. Soc. Perkin Trans. 2.

[98] Vishnumurthy K., Guru Row T.N., Venkatesan K. (1996). Studies in crystal engineering: Effect of fluorine substitution in crystal packing and topological photodimerization of styryl coumarins in the solid state. J. Chem. Soc. Perkin Trans. 2.

[99] Vishnumurthy K., Guru Row T.N., Venkatesan K. (1997). Studies in crystal engineering: Crystal packing, topological photodimerization and structure–reactivity correlations in fluoro-substituted styrylcoumarins. J. Chem. Soc. Perkin Trans. 2.

[100] Vishnumurthy K., Guru Row T.N., Venkatesan K. (1998). Unusual photodimerization of 7-fluoro-4-methylcoumarin and 6-fluoro-4-methylcoumarin in the solid state. Tetrahedron.

[101] Vishnumurthy K., Guru Row T.N., Venkatesan K. (1999). Studies in crystal engineering: Steering ability of fluorine in 4-styrylcoumarins. Tetrahedron.

[102] Mori Y., Matsumoto A. (2007). Photodimerization mechanism of bis(3,4,5-trifluorobenzyl) (*E*,*E*)-muconate in a columnar assembly in the crystalline state. Chem. Lett..

[103] Mori Y., Matsumoto A. (2007). Molecular stacking and photoreactions of fluorine-substituted benzyl muconates in the crystals. Cryst. Growth Des..

[104] Cozzi F., Ponzini F., Annunziata R., Cinquini M., Siegel J.S. (1995). Polar interactions between stacked π systems in fluorinated 1,8-diarylnaphthalenes: Importance of quadrupole moments in molecular recognition. Angew. Chem. Int. Ed. Engl..

[105] Cozzi F., Siegel J.S. (1995). Interaction between stacked aryl groups in 1,8-diarylnaphthalenes: Dominance of polar/π over charge-transfer effects. Pure Appl. Chem..

[106] Adams H., Blanco J.-L.J., Chessari G., Hunter C.A., Low C.M.R., Sanderson J.M., Vinter J.G. (2001). Quantitative determination of intermolecular interactions with fluorinated aromatic rings. Chem. Eur. J..

[107] Gung B.W., Patel M., Xue X. (2005). A threshold for charge transfer in aromatic interactions? A quantitative study of π-stacking interactions. J. Org. Chem..

[108] Reichenbächer K., Süss H.I., Hulliger J. (2005). Fluorine in crystal engineering—“the little atom that could”. Chem. Soc. Rev..

[109] Gung B.W., Xue X., Zou Y. (2007). Enthalpy (Δ*H*) and entropy (Δ*S*) for π-stacking interactions in near-sandwich configurations: Relative importance of electrostatic, dispersive, and charge-transfer effects. J. Org. Chem..

[110] Brock C.P., Naae D.G., Goodhand N., Hamor T.A. (1978). A statistical comparison of two determinations of the crystal structure of 2,3,4,5,6-pentafluorobiphenyl, a molecule forming mixed stacks in the solid state. Acta Crystallogr..

[111] Naae D.G. (1979). Biphenyl–perfluorobiphenyl; 1:1 molecular complex. Acta Crystallogr..

[112] Bartholomew G.P., Bazan G.C., Bu X., Lachicotte R.J. (2000). Packing modes of distyrylbenzene derivatives. Chem. Mater..

[113] Bartholomew G.P., Bu X., Bazan G.C. (2000). Preferential cocrystallization among distyrylbenzene derivatives. Chem. Mater..

[114] Feast W.J., Lövenich P.W., Puschmann H., Taliani C. (2001). Synthesis and structure of 4,4′-bis(2,3,4,5,6-pentafluorostyryl)stilbene, a self-assembling J aggregate based on aryl–fluoroaryl interactions. Chem. Commun..

[115] Bunz U.H.F., Enkelmann V. (1999). Structure elucidation, packing, and solid-state behavior of the eglinton - Galbraith dimer. Chem. Eur. J..

[116] Nishinaga T., Nodera N., Miyata Y., Komatsu K. (2002). Dehydro[12]- and -[18]annulenes fused with tetrafluorobenzene: Synthesis, electronic properties, packing structures, and reactivity in the solid state. J. Org. Chem..

[117] Ponzini F., Zagha R., Hardcastle K., Siegel J.S. (2000). Phenyl/pentafluorophenyl interactions and the generation of ordered mixed crystals: *sym*-Triphenethynylbenzene and *sym*-tris(perfluorophenethynyl)benzene. Angew. Chem. Int. Ed..

[118] Watt S.W., Dai C., Scott A.J., Burke J.M., Thomas R.L., Collings J.C., Viney C., Clegg W., Marder T.B. (2004). Structure and phase behavior of a 2:1 complex between arene- and fluoroarene-based conjugated rigid rods. Angew. Chem. Int. Ed..

[119] Smith C.E., Smith P.S., Thomas R.L., Robins E.G., Collings J.C., Dai C., Scott A.J., Borwick S., Batsanov A.S., Watt S.W., Clark S.J., Viney C., Howard J.A.K., Clegg W., Marder T.B. (2004). Arene-perfluoroarene interactions in crystal engineering: Structural preferences in polyfluorinated tolans. J. Mater. Chem..

[120] Coates G.W., Dunn A.R., Henling L.M., Ziller J.W., Lobkovsky E.B., Grubbs R.H. (1998). Phenyl-perfluorophenyl stacking interactions: Topochemical [2+2] photodimerization and photopolymerization of olefinic compounds. J. Am. Chem. Soc..

[121] Vishnumurthy K., Guru Row T.N., Venkatesan K. (2002). Fluorine in crystal engineering: Photodimerization of (1*E*,3*E*)-1-phenyl-4-pentafluorophenylbuta-1,3-dienes in the crystalline state. Photochem. Photobiol. Sci..

[122] Sonoda Y., Goto M., Tsuzuki S., Tamaoki N. (2007). Fluorinated diphenylpolyenes:Crystal structures and emission properties. J. Phys. Chem. A.

[123] Sonoda Y., Goto M., Tsuzuki S., Akiyama H., Tamaoki N. (2009). [2+2] Photodimerization and photopolymerization of diphenylhexatrienecrystals utilizing perfluorophenyl-phenyl stacking interactions. J. Fluorine Chem..

[124] Coates G.W., Dunn A.R., Henling L.M., Dougherty D.A., Grubbs R.H. (1997). Phenyl-perfluorophenyl stacking interactions: A new strategy for supermolecule construction. Angew. Chem. Int. Ed. Engl..

[125] Xu R., Gramlich V., Frauenrath H. (2006). Alternating diacetylene copolymer utilizing perfluorophenyl-phenyl interactions. J. Am. Chem. Soc..

[126] Liu J., Murray E.M., Young V.G. (2003). π-Stacking interactions in some crystalline cisoid *E*,*E*-1,4-diaryl-1,3-butadienes. Chem. Commun..

[127] Caronna T., Liantonio R., Logothetis T.A., Metrangolo P., Pilati T., Resnati G. (2004). Halogen bonding and π···π stacking control reactivity in the solid state. J. Am. Chem. Soc..

[128] Frontera A., Quiñonero D., Costa A., Ballester P., Deyà P.M. (2007). MP2 study of cooperative effects between cation–π, anion–π and π–π interactions. New J. Chem..

[129] Liu J., Boarman K.J. (2005). Regiospecific topochemical reactions controlled by trifluoromethyl directing groups. Chem. Commun..

[130] Liu J., Wendt N.L., Boarman K.J. (2005). Trifluoromethyl groups in crystal design of 1,4-diphenyl-1,3-butadienes for topochemical [2 + 2] photodimerization. Org. Lett..

[131] Jeannin O., Fourmigué M. (2006). Fluorine segregation in crystalline materials: Structural control and solid-state [2+2] cycloaddition in CF_3_-substituted tetrathiafulvalene derivatives. Chem. Eur. J..

[132] Hayashi S. (2004). wo-dimensional ^1^H spin-exchange NMR study of molecular arrangements in diphenylhexatrienes. Bull. Chem. Soc. Jpn..

[133] Turowska-Tyrk I., Grześniak K., Trzop E., Zych T. (2003). Monitoring structural transformations in crystals. Part 4. Monitoring structural changes in crystals of pyridine analogs of chalcone during [2+2]-photodimerization and possibilities of the reaction in hydroxy derivatives. J. Solid State Chem..

[134] Mahon M.F., Raithby P.R., Sparkes H.A. (2008). nvestigation of the factors favouring solid state [2 + 2] cycloaddition reactions; the [2 + 2] cycloaddition reaction of coumarin-3-carboxylic acid. CrystEngCommunity.

[135] Yang S.-Y., Naumov P., Fukuzumi S. (2009). Topochemical limits for solid-state photoreactivity by fine tuning of the π-π interactions. J. Am. Chem. Soc..

[136] Abdel-Mottaleb M.M.S., De Feyter S., Gesquière A., Sieffert M., Klapper M., Müllen K., De Schryver F.C. (2001). Photodimerization of cinnamate derivatives studied by STM. Nano Lett..

[137] Marubayashi N., Ogawa T., Hamasaki T., Hirayama N. (1997). A buffer zone in the crystal structure that governs the solid-state photodimerization of bulky olefins with the 1,4-dihydropyridine skeleton. J. Chem. Soc. Perkin Trans. 2.

[138] Gnanaguru K., Ramasubbu N., Venkatesan K., Ramamurthy V. (1984). Topochemical solid state photodimerization of non-ideally oriented monomers: 7-Chlorocoumarin and 7-methoxy-coumarin. J. Photochem..

[139] Bhadbhade M.M., Murthy G.S., Venkatesan K., Ramamurthy V. (1984). Topochemical dimerization of non-parallel double bonds: 7-Methoxycoumarin. Chem. Phys. Lett..

[140] Gnanaguru K., Ramasubbu N., Venkatesan K., Ramamurthy V. (1985). A Study on the photochemical dimerization of coumarins in the solid state. J. Org. Chem..

[141] Liang Y.-L., Fang J.-M., Chow T., Ho T.-I., Lee C.-R., Wang Y. (1994). Solution and solid-state photochemistry of 2-anilino-5-phenyl-2,4-pentadienenitriles. J. Org. Chem..

[142] Fonseca I., Hayes S.E., Bertmer M. (2009). Size effects of aromatic substitution in the *ortho* position on the photodimerization kinetics of α-*trans* cinnamic acid derivatives. A solid-state NMR study. Phys. Chem. Chem. Phys..

[143] Xu L.-P., Yan C.-J., Wan L.-J., Jiang S.-G., Liu M.-H. (2005). Light-induced structural transformation in self-assembled monolayer of 4-(amyloxy)cinnamic acid investigated with scanning tunneling microscopy. J. Phys. Chem. B.

[144] Swiatkiewicz J., Eisenhardt G., Prasad P.N., Thomas J.M., Jones W., Theocharis C.R. (1982). Phonon spectroscopy of photochemical reactions in organic solids: Photodimerization of 2-benzyl-5-benzylidenecyclopentanone and photopolymerization of 2,5-distyrylpyrazine. J. Phys. Chem..

[145] Ghosh M., Mandal T.K., Chakrabarti S., Misra T.N. (1998). Spectroscopic study of solid-state photoreaction in organic crystals: Photopolymerization of the dimethyl ester of *p*-phenylenediacrylic acid. J. Raman Spectrosc..

[146] Eckhardt C.J., Luty T., Peachey N.M. (1998). Collective interactions and solid state reactivity. Mol. Cryst. Liq. Cryst..

[147] Turowska-Tyrk I. (2001). Structural transformations in a crystal during the photochemical reaction of 2-benzyl-5-benzylidenecyclopentanone. Chem. Eur. J..

[148] Turowska-Tyrk I. (2003). Monitoring structural transformations in crystals. 5. A topotactic [2+2]-photodimerization reaction. Acta Crystallogr..

[149] Turowska-Tyrk I. (2004). Structural transformations in organic crystals during photochemical reactions. J. Phys. Org. Chem..

[150] Kim J.H., Matsuoka M., Fukunishi K. (1999). Selective topochemical photoreaction of crystallized 2,3-bis(2-phenylethenyl)-4,5-dicyanopyrazines. Chem. Lett..

[151] Natarajan A., Mague J.T., Venkatesan K., Ramamurthy V. (2005). Large molecular motions are tolerated in crystals of diamine double salt of *trans*-chlorocinnamic acids with *trans*-1,2-diaminocyclohexane. Org. Lett..

[152] Zheng S.-L., Pham O., Vande Velde C.M.L., Gembicky M., Coppens P. (2008). Competitive isomerization and dimerization in co-crystals of1,1,6,6-tetraphenyl-2,4-hexadiyne-1,6-diol and sorbic acid: A new look at stereochemical requirements for [2+2] dimerization. Chem. Commun..

[153] Robinson M.R., Wang S., Heeger A.J., Bazan G.C. (2001). A tetrahedral oligo(phenylenevinylene) molecule of intermediate dimensions: Effect of molecular shape on the morphology and electroluminescence of organic glasses. Adv. Func. Mater..

[154] Shirota Y. (2005). Photo- and electroactive amorphous molecular materials—molecular design, syntheses, reactions, properties, and applications. J. Mater. Chem..

[155] Nakanishi F., Nakanishi H., Hasegawa M., Yamada Y. (1975). Four-center type photopolymerization in the solid state. VII. Photochemical reaction of *m*-phenylene diacrylic acid dimethyl ester. J. Polym. Sci. Part A.

[156] Nakanishi H., Sasada Y. (1977). The crystal and molecular structure of dimethyl *m*-phenylenediacrylate. Bull. Chem. Soc. Jpn..

[157] Chakrabarti S., Maity A.K., Misra T.N. (1992). Spectroscopic study of solid-state photoreaction in organic crystal: Photopolymerization of dimethyl ester of α,α′-dicyano-*p*-phenylenediacrylic acid and diethyl ester of *p*-phenylenediacrylic acid. J. Polym. Sci. Part A.

[158] Minsk L.M., Smith J.G., Van Deusen W.P., Wright J.F. (1959). Photosensitive polymers. I. Cinnamate esters of poly(vinyl alcohol) and cellulose. J. Appl. Polym. Sci..

[159] Reiser A., Egerton P.L. (1979). The mechanism of crosslink formation in solid polyvinylcinnamate and related photopolymers. Photogr. Sci. Eng..

[160] Ichimura K. (1982). Preparation of water-soluble photoresist derived from poly(vinyl alcohol). J. Polym. Sci. Part A.

[161] Ichimura K., Watanabe S. (1982). Preparation and characteristics of photocrosslinkable poly(vinyl alcohol). J. Polym. Sci. Part A.

[162] Ichimura K., Oohara N. (1987). Photosensitive poly(methacrylates) having styrylpyridinium and styrylquinolinium groups. J. Polym. Sci. Part A.

[163] Skloss T.W., Haw J.F. (1994). Detection of cross-link formation by [2+2] photocycloaddition in poly(viny1 cinnamate) by ^13^C solid-state NMR. Macromolecules.

[164] Cockburn E.S., Davidson R.S., Pratt J.E. (1996). The photocrosslinking of styrylpyridinium salts via a [2 + 2]-cycloaddition reaction. J. Photochem. Photobiol. A Chem..

[165] Guo M., Xu Z., Wang X. (2008). Photofabrication of two-dimensional quasi-crystal patterns on UV-curable molecular azo glass films. Langmuir.

[166] Chung C.-M., Roh Y.-S., Cho S.-Y., Kim J.-G. (2004). Crack healing in polymeric materials via photochemical [2+2] cycloaddition. Chem. Mater..

[167] Li F., Zhuang J., Jiang G., Tang H., Xia A., Jiang L., Song Y., Li Y., Zhu D. (2008). A rewritable optical data storage material system by [2+2] photocycloreversion-photocycloaddition. Chem. Mater..

[168] Chung J.W., You Y., Huh H.S., An B.-K., Yoon S.-J., Kim S.H., Lee S.W., Park S.Y. (2009). Shear- and UV-induced fluorescence switching in stilbenic π-dimer crystals powered by reversible [2+2] cycloaddition. J. Am. Chem. Soc..

[169] Enkelmann V., Wegner G. Novak, Wagener K.B. (1993). Single-crystal-to-single-crystal photodimerization of cinnamic acid. J. Am. Chem. Soc..

[170] Enkelmann V., Wegner G., Novak K., Wagener K.B. (1994). Crystal-to-crystal photodimerizations. Mol. Cryst. Liq. Cryst..

[171] Takahashi S., Miura H., Kasai H., Okada S., Oikawa H., Nakanishi H. (2002). Single-crystal-to-single-crystal transformation of diolefin derivatives in nanocrystals. J. Am. Chem. Soc..

[172] Bučar D.-K., MacGillivray L.R. (2007). Preparation and reactivity of nanocrystalline cocrystals formed via sonocrystallization. J. Am. Chem. Soc..

